# A histone arginine methylation localizes to nucleosomes in satellite II and III DNA sequences in the human genome

**DOI:** 10.1186/1471-2164-13-630

**Published:** 2012-11-15

**Authors:** Daniel Capurso, Hao Xiong, Mark R Segal

**Affiliations:** 1 Department of Bioengineering and Therapeutic Sciences, San Francisco, CA, USA; 2 Department of Epidemiology and Biostatistics, University of California, San Francisco, CA, USA

**Keywords:** Epigenomics, Histone modifications, ChIP-Seq, Data pre-processing, Classification

## Abstract

**Background:**

Applying supervised learning/classification techniques to epigenomic data may reveal properties that differentiate histone modifications. Previous analyses sought to classify nucleosomes containing histone H2A/H4 arginine 3 symmetric dimethylation (H2A/H4R3me2s) or H2A.Z using human CD4^+^ T-cell chromatin immunoprecipitation sequencing (ChIP-Seq) data. However, these efforts only achieved modest accuracy with limited biological interpretation. Here, we investigate the impact of using appropriate data pre-processing —deduplication, normalization, and position- (peak-) finding to identify stable nucleosome positions — in conjunction with advanced classification algorithms, notably discriminatory motif feature selection and random forests. Performance assessments are based on accuracy and interpretative yield.

**Results:**

We achieved dramatically improved accuracy using histone modification features (99.0%; previous attempts, 68.3%) and DNA sequence features (94.1%; previous attempts, <60%). Furthermore, the algorithms elicited interpretable features that withstand permutation testing, including: the histone modifications H4K20me3 and H3K9me3, which are components of heterochromatin; and the motif TCCATT, which is part of the consensus sequence of satellite II and III DNA. Downstream analysis demonstrates that satellite II and III DNA in the human genome is occupied by stable nucleosomes containing H2A/H4R3me2s, H4K20me3, and/or H3K9me3, but not 18 other histone methylations. These results are consistent with the recent biochemical finding that H4R3me2s provides a binding site for the DNA methyltransferase (Dnmt3a) that methylates satellite II and III DNA.

**Conclusions:**

Classification algorithms applied to appropriately pre-processed ChIP-Seq data can accurately discriminate between histone modifications. Algorithms that facilitate interpretation, such as discriminatory motif feature selection, have the added potential to impart information about underlying biological mechanism.

## Background

Chromatin compaction is one of the critical factors regulating gene expression. The basic unit of chromatin, the nucleosome, consists of 147 base pairs (bp) of DNA wrapped around an octamer of histone proteins (H2A, H2B, H3, H4). Many histone post-translational modifications contribute to establishing compacted, transcriptionally repressed *hetero*chromatin (e.g., histone H3 lysine 9 trimethylation (H3K9me3)) or open, transcriptionally poised *eu*chromatin (e.g., H3K4me3) [1,2]. However, it is currently unknown why so many modifications — on at least 60 histone residues [3] — are necessary [3,4]. One possibility is that individual modifications have specialized properties, such as “indexing” classes of genomic elements [5]. Nevertheless, such discriminating properties remain largely unknown, as redundancy and enzyme promiscuity for non-histone targets have limited the amenability of histone modifications to genetic experimentation [6].

A potential solution to this problem is to apply supervised learning/classification techniques to high-throughput epigenomic data, such as chromatin immunoprecipitation sequencing (ChIP-Seq) data, for histone modificatons. Encouragingly, these approaches have had success in the related task of predicting the nucleosome occupancy of DNA sequences: they have elicited predictive features with biological (e.g., Rap1 transcription factor binding sites [7,8]) and biophysical (e.g., GC content, DNA propeller twist [7,9,10]) interpretations. Nevertheless, attempts to apply classification techniques to histone modifications have been less forthcoming. This is, in part, because such analyses require less readily available datasets, which correspond to many ChIP-Seq experiments in the same cell type. As notable exceptions, Barski et al. [11] have generated a ChIP-Seq dataset for 20 histone methylations and the histone variant H2A.Z in human CD4^+^ T cells, and Wang et al. [12], of the same research group, have generated a similar dataset for 18 histone acetylations. A recent study by Gervais and Gaudreau [13] applied classification techniques to histone modifications using these datasets.

In particular, Gervais and Gaudreau [13] attempted to predict whether a nucleosome contains histone H2A.Z or H2A/H4 arginine 3 symmetric dimethylation (H2A/H4R3me2s; the authors refer to this as just “H2A”, though it is a methylated form [14]). Importantly, these two classes are likely mutually exclusive: H2A.Z lacks the R3 methylation site and localizes near active transcription start sites [1], while H2A/H4R3me2s localizes with repressed heterochromatin [3]. The authors [13] first performed classification with histone modification features (co-localization with 37 other modifications from ChIP-Seq) and, then, with DNA sequence features (frequency of 6-mers in 147 bp nucleosome-bound DNA sequences). However, these analyses only achieved modest prediction accuracies of 68.3% and <60%, respectively (here, a trivial classifier would have an accuracy of 50%) [13]. Furthermore, there was limited biological interpretation for histone modification features and no interpretation for DNA sequence features [13].

A partial explanation for this modest performance may be insufficient data pre-processing. Gervais and Gaudreau [13] used *raw*, aligned (25 bp) ChIP-Seq reads, and simply extended these to 147 bp to generate what they consider to be nucleosome-bound DNA sequences. However, this approach is problematic. Because ChIP-Seq is only a slight enrichment (not a purification) for sequences bound to the protein of interest [15], it is notoriously noisy. The majority (estimates upward of 90% [16]) of ChIP-Seq reads are instead from the background. Therefore, we, and others [15,17,18], advocate using position- (peak-) finding algorithms, such as Nucleosome Positioning from Sequencing (NPS) [17] (see *Methods*), that identify stable nucleosome positions with statistically significant enrichment over background, prior to analysis. Here, *stable* nucleosomes can be defined as those that are located at roughly the same chromosomal position across a population of cells and can therefore generate a signal peak when ChIP-Seq reads are aligned. Such nucleosomes are also referred to as being relatively well *positioned* or *phased*, and there is evidence for their regulatory importance [1,16]. While using stable nucleosome positions might limit the analysis to a subset of nucleosomes (and thus influence interpretation), we still believe this approach is preferable to using raw, aligned reads — of which only a small minority were likely even bound to the nucleosomes of interest. This approach of using stable nucleosomes was also utilized in a recent study [19].

Aside from the handling of signal and background, the approach of Gervais and Gaudreau [13] might not adequately control for systematic biases present in ChIP-Seq data. First, because of PCR amplification bias it may be advisable to collapse duplicate reads prior to analysis [15,18]. This is especially the case for datasets such as Barski et al. [11] and Wang et al. [12] where sequencing depth is relatively low, such that there is a lower likelihood of sequencing independently-precipitated fragments with the same start site (as future datasets begin to have much higher sequencing depth, more refined alternatives to read deduplication will be valuable). Indeed, even for stable nucleosomes, the positioning is often blurry, with nucleosomes not having precisely the same start site across cells [20]. In addition, because coverage and the ability to detect peaks vary with sequencing depth, ChIP-Seq experiments need to be normalized for the number of reads [18]. Refined normalization approaches are emerging [21] for ChIP-Seq datasets that contain a mock immunoprecipitation (IP) sample; however, for otherwise rich ChIP-Seq datasets that lack such a mock IP, including [11] and [12], we believe data should still be normalized for the number of reads, in the absence of a more delicate approach for this type of data (see Discussion).

Here, we employ appropriate ChIP-Seq data pre-processing and sequence-customized, or otherwise advanced, algorithms to investigate their impact on the accuracy and interpretability of classifying nucleosomes containing H2A/H4R3me2s or H2A.Z. For data pre-processing, we perform deduplication, normalization, and position-finding. Further, for DNA sequence-based classification, we utilize the recently developed Discriminatory Motif Feature Selection (DMFS) [22], which, in addition to achieving impressive accuracy, emphasizes interpretability, unlike so-called “black-box” classifiers. Specifically, DMFS elicits a small set of a priori discriminatory features (motifs) on a subsequently withheld data partition. This eliminates many noise features, which can comprise prediction and interpretation [23], and loosens restrictive feature length prescriptions (e.g., 6-mers in [13]), which could otherwise fail to generate key, longer features. For classification based on histone modification features, we utilize an ensemble method, random forests [24], which has been widely demonstrated to improve on individual classification trees [24,25], as were deployed by Gervais and Gaudreau [13]. Finally, we perform extensive downstream analysis. Importantly, in addition to achieving dramatically improved accuracies, our classification algorithms elicit predictive, interpretable features that are consistent with recent biochemical findings [26].

## Results

We pre-processed the Barski et al. [11] ChIP-Seq dataset for 20 histone methylations and H2A.Z to reduce bias. The percentage of duplicate reads in each experiment ranged from 2.1% to 25.1% (*median* = 5.6%), suggesting the potential for substantial PCR bias in some of the samples. We therefore collapsed duplicate reads into single reads. Additionally, the number of unique reads in the experiments varied by more than 3-fold, indicating the potential for considerable sequencing depth variation (and thus coverage bias) across the raw samples. We therefore normalized experiments for sequencing depth by down-sampling to the lowest number of unique reads observed (see *Methods*).

Using this filtered data, we identified stable nucleosome positions as signal peaks with statistically significant enrichment over the background by applying NPS [17] (see *Methods*). This yielded 1845 and 46235 stable nucleosomes containing H2A/H4R3me2s and H2A.Z, respectively (Additional file 1: Table S1). Next, we down-sampled H2A.Z nucleosomes to match the number of H2A/H4R3me2s nucleosomes for two reasons. First, this creates a balanced dataset for classification (i.e., where a trivial classifier has an accuracy of 50%) and thus yields accuracies directly comparable to those of [13] (who performed analogous down-sampling). Indeed, using “class-imbalanced” data can result in a classifier that is biased toward the larger class [27]; in the case of high-dimensional data, down-sampling the larger class is preferable to over-sampling the smaller class [27]. Second, down-sampling emphasizes features associated with H2A/H4R3me2s, which is relatively under-studied compared to H2A.Z. An added benefit of this approach is its reduction of the computational burden. All reported performance results are the mean of (cross-validated or out-of-bag) performance summaries over 10 different random down-samplings of H2A.Z nucleosomes, to ensure our balanced approach did not bias the results.

### Classification using histone modification features

The presence of one type of histone modification in a nucleosome can increase or decrease the likelihood of a second type [2]. Therefore, to identify such potential interactions, we attempted to discriminate between stable nucleosomes containing H2A/H4R3me2s or H2A.Z by using the co-localization with 19 other histone methylations and 18 histone acetylations (Additional file 1: Table S2) as features for classification. For each stable nucleosome, we generated an array of length 37 (for 37 feature modifications), where each entry is the number of deduplicated sequence reads for a feature modification that map within the nucleosome boundaries in a strand-specific manner (see *Methods*). The motivation for using deduplicated sequence read counts for scoring overlap with feature modifications is that it results in a richer (i.e., less sparse) matrix than scoring binary overlap with stable nucleosomes for the feature modifications. We still use stable nucleosomes, however, for the outcome modifications (H2A/H4R3me2s, H2A.Z) and in downstream analyses.

**Figure 1:**
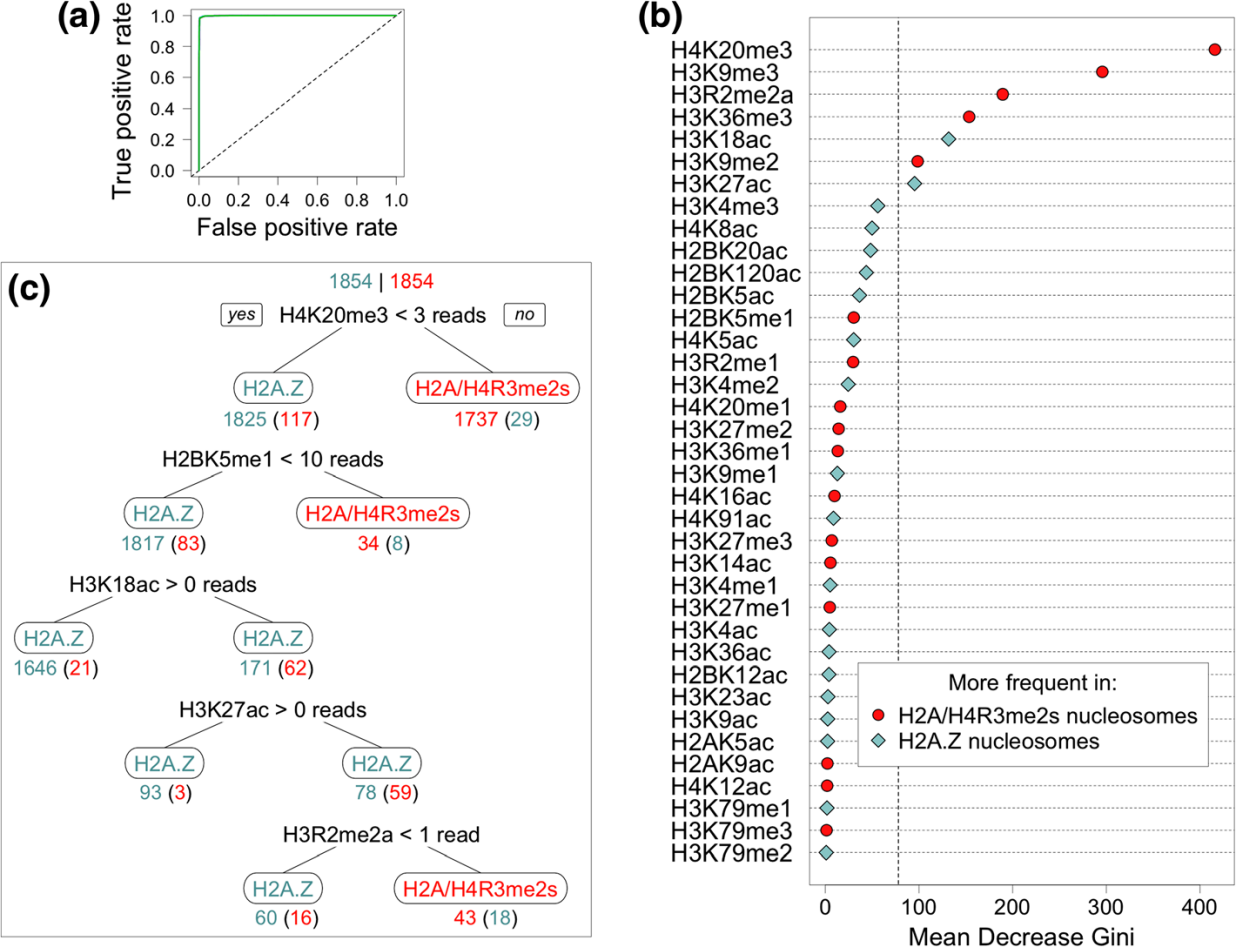
**Classifying stable nucleosomes containing H2A/H4R3me2s or H2A.Z using histone modification features.** (**a**) Receiver Operating Characteristic (ROC) curve, demonstrating classifier performance. (**b**) Random forests feature importance by mean decrease in Gini index. Features have a higher frequency in H2A/H4R3me2s nucleosomes (*red*) or H2A.Z nucleosomes (*blue*). The dashed, vertical line shows the estimated (permutation-based) significance threshold after multiple testing correction. (**c**) A classification tree with splits (*no borders*) and leaves (*borders*), below which is the number of nucleosomes classified correctly and, in parentheses, incorrectly at that stage. Leaves show the predicted class labels of nucleosomes partitioned there. Splits show the condition that best separates the data. Branch labels indicate the directions in which the split condition is true (“*yes*”) and false (“*no*”).

We attained highly accurate random forests (see *Methods*) prediction performance using histone modification features, with an accuracy of 99.0% ± 0.1% and an area under the Receiver Operating Characteristic curve (auROC) of 0.999 ± 0.0002 (Figure 1a). This is a substantial improvement over the corresponding accuracy of 68.3% that Gervais and Gaudreau [13] report. To determine which features were “driving” the classification, we evaluated random forests feature importance by mean decrease in Gini index (MDG; Figure 1b; see *Methods*). Several features ranked prominently and withstood estimation of statistical significance by permutation testing (see *Methods*): H4K20me3, H3K9me3, H3R2me2a, H3K36me3, H3K18ac, H3K9me2, and H3K27ac had a permutation *p* < 1e-05 (Bonferroni-adjusted *p* < 3.7e-04; Figure 1b). The remaining histone modification features were not significant.

To further explore how these features relate to H2A/H4R3me2s, we built a single classification tree (Figure 1c) [28], which, compared to the random forests ensemble of trees, may more readily reveal interpretable rules, albeit at the cost of decreased classification accuracy. Consistent with the random forests feature importance ranking, the feature that best separated the data in the single tree is H4K20me3 (Figure 1c). Indeed, 1737 out of 1854 stable nucleosomes containing H2A/H4R3me2s were classified at the first split, based on overlapping with greater than two deduplicated, H4K20me3 sequence reads (with a misclassification rate of only 1.67%). Three of the four remaining splits were also based on features that were had significant random forests feature importances (H3K18ac, H3K27ac, and H3R2me2a; H2BK5me1 did not have a significant random forests feature importance, yet was the basis for the second split). H3K9me3, which had the second highest random forests feature importance, was not the basis for a split in the single tree; however, this may occur if, for example, the stable H2A/H4R3me2s nucleosomes that overlap with H3K9me3 are a subset of those that overlap with H4K20me3 (and so they are already classified at the first split).

Encouragingly, the top two modifications by random forests feature importance, H4K20me3 and H3K9me3, are more frequent in stable nucleosomes containing H2A/H4R3me2s than those containing H2A.Z (Figure 1b). Because H4K20me3 and H3K9me3 have been shown to contribute to the formation of heterochromatin [1,2] – which is where H2A/H4R3me2s localizes — this initial finding supports the biological relevance of our classifier.

### Classification using DNA sequence features

DNA sequence likely influences the genome-wide distribution of histone modifications, as sequence-specific transcription factors and microRNAs can bind and recruit histone-modifying enzymes [29]. Thus, we used DNA sequence motifs as features for classifying H2A/H4R3me2s and H2A.Z nucleosomes for two reasons: first, to identify such potential targeting sequences, and second, to identify classes of genomic elements that the histone modification potentially regulates. Using DMFS [22], we identified <300 a priori discriminatory motifs with lengths between 5 and 10 bp from a subsequently withheld partition of the data (see *Methods*).

**Figure 2:**
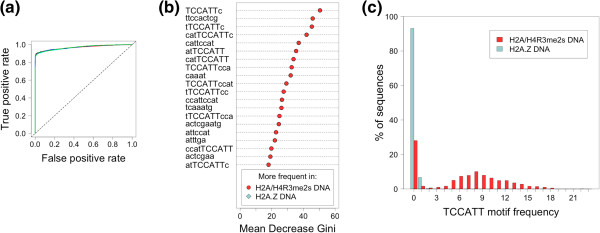
**Classifying stable nucleosomes containing H2A/H4R3me2s or H2A.Z using DNA sequence features.** (**a**) Receiver Operating Characteristic (ROC) curve, demonstrating classifier performance. (**b**) Random forests feature importance by mean decrease in Gini index. Features have a higher frequency in H2A/H4R3me2s nucleosomal DNA (*red*) or H2A.Z nucleosomal DNA (*blue*). (**c**) Frequency histogram of the number of occurrences of the motif TCCATT in H2A/H4R3me2s nucleosomal DNA (*red*) or H2A.Z nucleosomal DNA (*blue*).

As above, we attained highly accurate random forests prediction performance using DNA sequence features (discriminatory motifs), with an accuracy of 94.1% ± 0.3% (auROC = 0.968 ± 0.001; Figure 2a). This is a dramatic improvement over the corresponding accuracy of <60% that Gervais and Gaudreau [13] report. We next evaluated random forests feature importance by MDG (see *Methods*). The top 20 features (Figure 2b), all of which occur more frequently in DNA corresponding to stable H2A/H4R3me2s nucleosome positions, withstand estimation of statistical significance by permutation testing, with permutation *p* < 1e-05 (Bonferroni-adjusted *p* < 2.7e-03). Interestingly, 12 of these 20 sequence features contain the motif TCCATT (Figure 2b). We therefore analyzed the frequency distribution of the number of occurrences of this motif in the DNA sequences corresponding to stable nucleosome positions (Figure 2c, Additional file 1: Table S3). Indeed, while the motif TCCATT is present in only ~7% of stable H2A.Z nucleosomal DNA sequences (*max* = 3 occurrences per sequence), it is present in ~72% of stable H2A/H4R3me2s nucleosomal DNA sequences (*max* = 23 occurrences per sequence; *median =* 7*;* Figure 2c). That this 6-mer occurs so abundantly in many of the stable H2A/H4R3me2s nucleosomal DNA sequences is suggestive of it being a repetitive element, or component thereof – an observation we explore in downstream analysis.

For thoroughness, however, we first performed a combined classification that utilized histone modification features *and* DNA sequence features. This resulted in a classification accuracy of 98.6% ± 0.1% (auROC = 0.999 ± 0.0002). Feature importance analysis by MDG yielded many of the same top features as in the separate classifications, namely: H4K20me3, H3K9me3, H3R2me2a, H3K36me3, and sequences containing the motif TCCATT.

### Downstream feature analysis

Having elicited important, predictive features (particularly H4K20me3, H3K9me3, and the sequence motif TCCATT), we pursued downstream analysis in an attempt to determine how they relate functionally to H2A/H4R3me2s. First, given the abundant occurrence of the motif TCCATT, we referenced the DNA sequence composition of repetitive elements in the human genome. Indeed, TCCATT is part of the consensus sequence of satellite II and III DNA (Table 1) [30,31], which are types of transcriptionally competent, tandem repetitive elements located primarily in pericentromeric regions [30].

**Table 1:** Satellite II and III DNA consensus sequences

**Satellite type**	**Consensus sequence**
satellite II DNA	[(atTCCATTcg)_2_ + (atg)_1–2_]_n_
satellite III DNA	[(ATTCC)_7–13_ + (ATTcgggttg)_1_]_n_

Subscripts indicate the number of occurrences of a subsequence in the consensus sequence. The motif TCCATT is displayed in uppercase. For satellite III DNA, the motif also appears when two instances of the first subsequence are juxtaposed. Adapted from [30,31].

**Figure 3:**
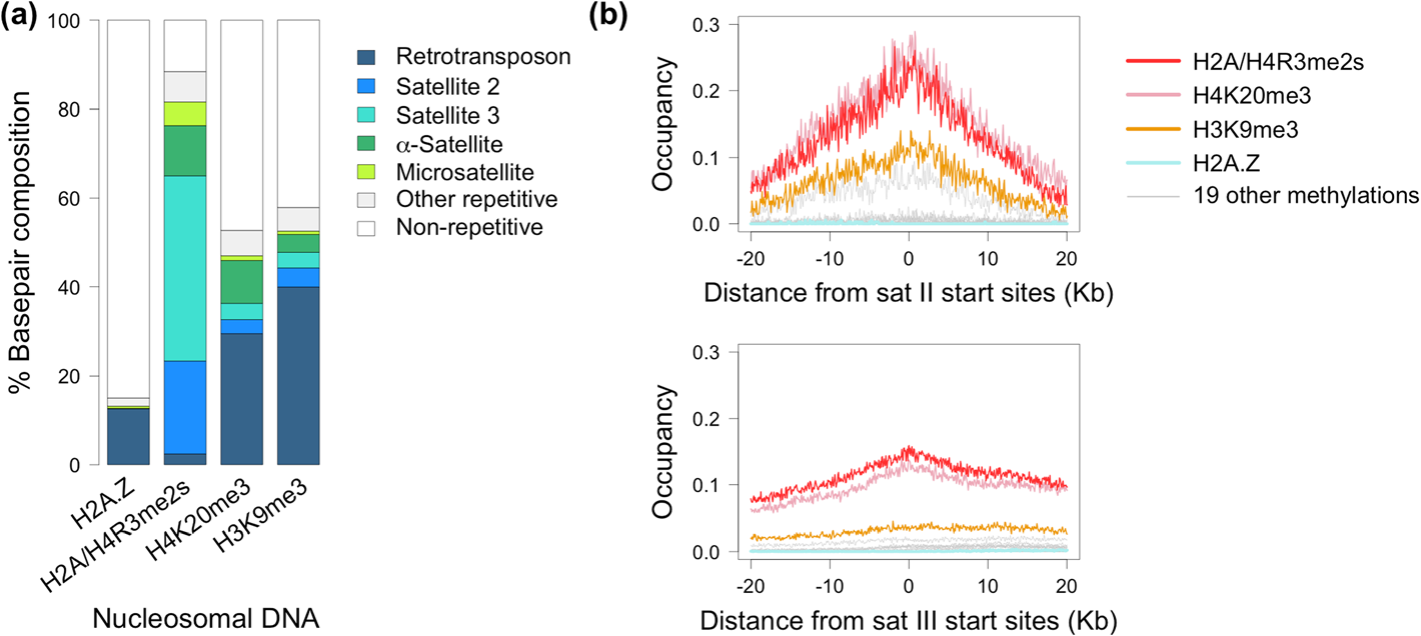
**Relationship between stable nucleosomes containing histone modifications and satellite II and III DNA sequences.** (**a**) The percentage contributions of types of repetitive elements to the total DNA sequence bound to nucleosomes containing the indicated histone modification. (**b**) The fraction of start site -aligned satellite II (*upper*) or III (*lower*) DNA sequences occupied by stable nucleosomes containing the indicated histone modification.

To determine if satellite II and III DNA are the source of the TCCATT motif detected, we analyzed the percentage of the total DNA sequence bound to stable nucleosomes containing various histone modifications that is annotated as satellite II and III DNA (or other repetitive elements; Figure 3a). Indeed, around 63% of the total DNA sequence bound to stable H2A/H4R3me2s nucleosomes is satellite II and III DNA, while none of the stable H2A.Z nucleosome -bound DNA is (Figure 3a). Satellite II and III DNA also contribute to the DNA sequence bound to stable nucleosomes containing H4K20me3 or H3K9me3, though they comprise a lower percentage (around 7% and 8%, respectively; Figure 3a). Thus, stable H2A/H4R3me2s nucleosomal DNA is enriched for TCCATT motifs derived from satellite II and III DNA. As an interesting aside, we found that a substantial portion of the DNA bound to stable nucleosomes containing H4K20me3 or H3K9me3 is retrotransposons; this is not the case for stable nucleosomes containing H2A/H4R3me2s.

Finally, we explored further the relationship between satellite II and III DNA and various histone modifications. For each histone modification, we calculated *occupancy*[32] over aligned satellite II (or III) DNA sequences, where occupancy is defined as the fraction of sequences at a position that are bound to a stable nucleosome containing that histone modification (see *Methods*). We found that H2A/H4R3me2s and H4K20me3 had the highest occupancy over satellite II DNA sequences (0.266 and 0.289, respectively) and satellite III DNA sequences (0.159 and 0.142, respectively). H3K9me3 followed closely with occupancies of 0.140 and 0.045 over satellite II and III DNA, respectively. On the other hand, H2A.Z and 18 other histone methylations in the Barski et al. [11] datatset had no or almost no occupancy over these satellites (1 methylation, H3R2me2a, had low occupancy). These findings are depicted in Figure 3b.

Thus, downstream analysis functionally relates the elicited features to H2A/H4R3me2s and to each other: H2A/H4R3me2s, H4K20me3, and H3K9me3 all occur on stable nucleosomes in satellite II and III DNA sequences, from which the motif TCCATT is derived. These interactions are consistent with recent biochemical experimental results, a point we return to in the Discussion.

## Discussion

Emerging, high-throughput epigenomic data, including ChIP-Seq data, may provide insight into mechanisms of chromatin structure and gene regulation. However, realizing the full potential of this data requires a computational framework that reduces bias, maximizes algorithm accuracy, and elicits predictive and biologically interpretable features. To this end, we classified nucleosomes containing H2A/H4R3me2s or H2A.Z, as in [13], but instead employed appropriate data pre-processing and advanced classification algorithms, resulting in greatly improved accuracy and interpretative yield.

Indeed, interpretation of ChIP-Seq is challenging because of the magnitude and complexity of the data (issues of quality and pre-processing, aside). This is particularly true when comparing multiple histone modifications (or transcription factors). Encouragingly, approaches aiming to improve ChIP-Seq interpretation, albeit not directly applicable to our analyses, appear in the recent literature. For example, Fernandez et al. [33] use a genetic algorithm to identify the optimal number of histone modification profiles to combine to identify transcriptional enhancers, while Beck et al. [34] aim to improve ChIP-Seq interpretation by incorporating information about peak shape via linear predictive coding.

In light of these challenges, and given the problems with enumerative feature approaches (e.g., all 6-mers; discussed in detail below), we decided to employ a recently devised pipeline for sequence-based classification, DMFS [22], that focuses on feature interpretation. DMFS elicits a small set of a priori discriminatory features (motifs) using a subsequently withheld data partition. Using DMFS, we evaluated a feature length range between 5 and 10 bp by eliciting < 300 a priori discriminatory motifs. In contrast, evaluating this length range with enumerative approaches would require a burdensome, if not prohibitive, Σ 4^k^ = 1397760 features. Thus, feature length often needs to be highly restricted for enumerative approaches, which can then fail to elicit longer, potentially important (interpretable) features. Even with feature length prescriptions, enumerative approaches still employ multitudes of noise features, which can degrade performance [23] and complicate determination of feature importance and interpretation. Thus, using DMFS to eliminate univariately unimportant features at the outset has advantages; however, it can miss features whose effects are strict (second or higher order) interactions.

Some attempts have been made to improve interpretation of enumerative feature classification. Most existing enumerative techniques rely heavily on support vector machine (SVM) classifiers that employ sophisticated, problem-specific kernels, notably the spectrum kernel [36] and variants thereof [37,38], such as the so-called “blended spectrum” kernel used previously [13] to analyze the data considered here. Determining feature importance for such approaches is arguably very challenging (it is challenging, in general, for SVMs), given inherent feature dependencies (overlaps at neighboring positions) and kernel complexity. Some inventive methods have been developed to address these issues [39,40]. Nevertheless, these methods are necessarily constrained: input sequences need to be the same length and only select SVM kernels are supported. Thus, another advantage of the DMFS approach is that it provides a modular, all-purpose, pipeline applicable to any (binary) classification problem with any sequence inputs.

In the current study, we employed DMFS for sequence-based classification using pre-processed data. For the sake of comparability, we also tried applying DMFS to *raw*, aligned, extended ChIP-Seq reads as used in [13], which resulted in a classification accuracy similar to that of Gervais and Gaudreau [13]. Thus, while DMFS provided the benefits of ready interpretation, modularity, and computational efficiency, the improvements in performance that we achieved are largely attributable to data pre-processing. Indeed, several authors [15,18] have advocated ChIP-Seq data pre-processing based on observations of bias and extensive background reads. Peak-finding methods have also been specifically designed for histone modification ChIP-Seq data: SICER [41] identifies broad chromatin domains enriched for a histone modification, while NPS [17] identifies individual, stable nucleosomes that contain a histone modification. Our study is valuable in that it demonstrates empirically the gains in classification performance that result from ChIP-Seq data pre-processing, thus substantiating the advocacy thereof.

Another valuable aspect of our study is that the identified features are consistent with recent biochemical experimental results. Our classification approaches identified the motif TCCATT (derived from satellite II and III DNA sequences) and the histone modifications H4K20me3 and H3K9me3 as predictive of H2A/H4R3me2s nucleosomes. Consistent with this, Zhao et al. [26] recently demonstrated that H4R3me2s provides a direct binding site for the DNA methyltransferase (Dnmt3A) that methylates satellite II and III DNA [42-44]. The enzyme that mediates H3K9me3 also interacts directly with Dnmt3A [45]. Furthermore, the proper occurrence of H4K20me3 and H3K9me3 has been shown to be partially dependent on Prmt5, the enzyme that mediates H2A/H4R3me2s [46]. Interestingly, the aberrant expression of satellite II and III DNA, which is observed in senescent cells [47] and cancers [44,48], may promote genomic instability via chromosomal rearrangements [49]. Thus, our finding that H2A/H4R3me2s, H4K20me3, and H3K9me3 occur in stable nucleosomes in satellite II and III DNA sequences genome-wide may be consequential in terms of understanding how these genomic elements are normally repressed in healthy, differentiated tissue.

In future work, we will extend our analyses to classifying the 19 other histone modifications in the Barski et al. [11] dataset. This could be realized using an iterative one-against-all approach, which would be more high-throughput (albeit at the potential cost of diluting discriminatory signals), or using a targeted, biologically motivated approach. With respect to the latter, of particular interest would be discriminating between histone modifications that localize with facultative (e.g., H3K27me3) and constitutive (e.g., H3K9me3) heterochromatin. Indeed, DNA elements capable of recruiting the facultative heterochromatin machinery have not been identified in the human genome so far, though they have been in the *Drosophila* genome (i.e., Polycomb Response Elements [35]). Additionally, we will explore the impact of alternative ChIP-Seq normalization approaches, including some more refined, emerging methods [21]. However, because such methods often rely on a mock immunoprecipitation (IP) sample, which many otherwise rich ChIP-Seq datasets lack (including Barski et al. [11]), it would be worthwhile to pursue developing a method for identifying the background in datasets with multiple experimental IPs but no mock IP. Similarly, it would be a great advance to develop an algorithm that could identify and remove read buildups that correspond to PCR amplification bias without collapsing “biological” duplicate reads – especially as the latter will be common in newer datasets with very high sequencing depth. Finally, we could pursue, though more ambitious, developing an algorithm for multi-class classification with a similarly discriminatory framework [22].

## Conclusions

Our study demonstrates that applying advanced classification algorithms to appropriately pre-processed ChIP-Seq data results in greatly improved prediction accuracy and feature interpretative yield in genome-wide discrimination between histone modifications. The discriminatory motif feature selection approach that we employed has the added potential to facilitate interpretation of the biological mechanism underlying the classifier performance. Finally, and perhaps most importantly, the findings presented here demonstrate that statistical/machine learning analyses of epigenomic data can identify interpretable, biologically meaningful properties of histone modifications, which have been difficult to study by traditional genetic experimentation.

## Methods

### ChIP-Seq data pre-processing

The Barski et al. [11] ChIP-Seq dataset for 20 histone methylations and H2A.Z in human CD4^+^ T cells was downloaded as BED files of mapped ChIP-Seq reads from: <http://dir.nhlbi.nih.gov/papers/lmi/epigenomes/hgtcell.aspx>. In each sample, duplicate reads were collapsed into single reads to eliminate PCR amplification bias [15,18]. Samples were normalized for unique read number via down-sampling, in order to eliminate bias from sequencing depth variation [18]. Stable nucleosomes with statistically significant enrichment over the background were identified, using NPS [17], for each of the 20 histone methylations and H2A.Z.

NPS extends reads in the 3' direction to 150 bp, corresponding to the length of the MNase-digested mononucleosomal DNA [11,17]. NPS then employs signal sampling and wavelet denoising to improve signal resolution and reduce background, and Laplacian of Gaussian methods to detect peak edges [17]. We only accepted peaks that pass quality control filtering and statistical significance testing, as in [17], to reduce false positives. Specifically, peaks must have had a width 80 bp ≤ *w* ≤ 250 bp, a strand ratio *s* ≤ 3, and a significant number of reads (Poisson *p* ≤ 1e-05). For each such nucleosome peak, we extended the midpoint to 147 bp for use in classification.

### Classification/Feature elicitation

H2A.Z nucleosomes were down-sampled to match the number of H2A/H4R3me2s nucleosomes to create a balanced classification scheme [27]. All performance evaluations are based on the mean of ten random samples of H2A.Z nucleosomes to ensure sampling did not impact the results. Classification was performed using random forests [24], an algorithm that averages over an ensemble of classification trees. Briefly, each tree is constructed from a bootstrap sample of the data. Unlike conventional trees, where each node is split using the overall most predictive feature, each node in random forest trees is split using the most predictive feature from a subset of features randomly sampled at that node. This additional injection of randomness serves to de-correlate trees in the ensemble, so that subsequent averaging over the ensemble more effectively decreases prediction variance and thereby improves prediction performance [25]. An unbiased estimate of the prediction error rate is obtained as follows: first, for each tree in the ensemble, classify the data points not included in the bootstrap sample for that tree (so-called out-of-bag (OOB) data); then, average the predictions across all trees where a given data point was OOB [24,25,50].

Random forests have two primary parameters: for the number of trees, we used *n*_*tree*_ = 500; and for the subset of features sampled at each node, we used the default classification value *m*_*try*_ = sqrt(*p*), where *p* is the number of features. Compared to other classifiers, random forests have the advantage of being relatively resistant to overfitting and relatively insensitive to parameter tuning, as long as *n*_*tree*_ is sufficiently large [24,50]. All reported area under the Receiver Operating Characteristic curve (*auROC*) values are for random forests, though, for thoroughness, classifications were repeated using support vector machines (SVMs); comparable results were obtained. Fitting of both random forests and SVMs made recourse to the corresponding R packages [50,51] and to the ROCR package [52].

Classification was performed using two distinct feature types: histone modification features and DNA sequence features. For histone modification features, we used the 19 histone methylations remaining in the Barski et al. [11] dataset, as well as 18 histone acetylations from the Wang et al. [12] dataset, which was generated by the same research group and in the same cell type. The latter dataset was downloaded from: <http://dir.nhlbi.nih.gov/papers/lmi/epigenomes/hgtcellacetylation.aspx>. To create the overlap matrix, an array of length 37 (for 37 histone modification features) was created for each stable H2A/H4R3me2s or H2A.Z nucleosome. Each entry in the array indicates the number of de-duplicated sequence reads for the given feature modification that co-localize with the stable nucleosome boundaries in a strand-specific manner. Specifically, to be scored: ‘+’ strand feature reads must map within ± 50 bp of the 5' stable nucleosome boundary, and ‘-’ strand feature reads must map within ± 50 bp of the 3' stable nucleosome boundary.

To generate DNA sequence features, we used DMFS: <https://bitbucket.org/haoxiong/dmfs-code/>[22]. DMFS elicits a small set of a priori informative motifs that discriminate between positive (here, H2A/H4R3me2s) and negative (here, H2A.Z) classes. Unlike enumerative (e.g., all 6-mers) approaches, DMFS avoids the generation of abundant noise features, which can compromise prediction and interpretation [23]. Additionally, it allows longer, potentially informative features to be evaluated. To avoid data reusage, DMFS requires an additional level of data partitioning, utilizing a *discovery* set for initial discriminatory motif finding and a *classification* set for subsequent random forest (or SVM) analysis. For the fraction of nucleosomal sequences allocated to the discovery set, we used the recommended value *f* = 0.2 [22]; we ultimately evaluated five instances of the data being randomly partitioned as such, to ensure partitioning did not impact the results. A key component of the DMFS pipeline is the tool employed for eliciting discriminatory motifs. We used the default tool – Wordspy [53,54] – selected in view of its impressive performance in benchmarking studies [54]. Remaining DMFS parameter settings were: minimum motif length *l* = 5, maximum motif length *m* = 10 (with both DNA strands being searched); and at most *M* = 2 mismatches, when aligning elicited motifs to classification set sequences.

### Feature importance and downstream analysis

To identify the most individually predictive features, random forest feature importance was assessed using the mean decrease in Gini index (MDG). Briefly, the Gini index is a measure of statistical impurity. Every time a node is split in a tree, the daughter nodes become more homogenous and, thus, have a lower Gini index than the parent node. A robust measurement of feature importance can obtained as follows: for each feature, average across all random forest trees the decrease in Gini index that results from splitting a node on that feature [25]. Permutation testing was performed to estimate the statistical significance of variable importance: MDG scores were compared to the distribution of scores from 100,000 classifications using data with permuted class labels.

Downstream analysis was performed for a motif found in many of the elicited sequence features. The genomic coordinates of repetitive DNA sequences were downloaded from the RepeatMasker track of the Table Browser [55] of the UCSC Genome Browser (build hg18). Based on Repbase Update [56] annotations, satellite II DNA (*repName* = HSATII) and satellite III DNA (*repName* = (CATTC)n, (GAATG)n) coordinates were extracted. For each histone modification, we calculated the percentage of its total stable nucleosome-bound DNA sequence that consists of satellite II or III DNA. Additionally, for each histone modification, we calculated its *occupancy* along satellite II DNA, or satellite III DNA, sequences aligned by start site — where *occupancy*[32] is defined as the fraction of sequences bound to a stable nucleosome, in this context, with the histone modification.

## Abbreviations

ChIP-Seq: chromatin immunoprecipitation sequencing; bp: base pairs; H2A, H2B, H3, and H4: histone proteins H2A, H2B, H3, and H4; H2A.Z: histone variant protein H2A.Z; R: arginine; K: lysine; me: methylation; me2: dimethylation; me2s: symmetric dimethylation; me3: trimethylation; auROC: area under the Receiver Operating Characteristic curve; MDG: mean decrease in Gini index; SVM: support vector machines; DMFS: Discriminatory Motif Feature Selection; NPS: Nucleosome Positioning from Sequencing.

## Competing interests

The authors declare that they have no competing interests.

## Authors’ contributions

DC and MRS conceived the study. MRS designed the study. DC, HX, and MRS performed the analyses. DC and MRS wrote the manuscript. DC, HX, and MRS proofread and approved the manuscript.

## Supplementary Material

Additional file 1 **Table S1.** Number of sequence reads (or stable nucleosomes) for histone methylations and H2A.Z at each data pre-processing step. **Table S2.** Number of sequence reads for histone acetylations at each data preprocessing step. **Table S3.** Percentage of sequence reads at each data pre-processing step that contain the motif “TCCATT”. Click here for file
