# Supplementary material for: A histone arginine methylation localizes to nucleosomes in satellite II and III DNA sequences in the human genome
Source: BMC Genomics. 2012 Nov 15;13:630. doi: 10.1186/1471-2164-13-630 (PMC3559892; doi:10.1186/1471-2164-13-630)
Supplement: Additional file 1 — Table S1. Number of sequence reads (or stable nucleosomes) for histone methylations and H2A.Z at each data pre-processing step. Table S2. Number of sequence reads for histone acetylations at each data preprocessing step. Table S3. Percentage of sequence reads at each data pre-processing step that contain the motif “TCCATT”. [file 1471-2164-13-630-S1.pdf]

Supplementary data for manuscript:

A histone arginine methylation localizes to nucleosomes in satellite II and III DNA sequences in the human genome

Daniel Capurso[1] , Hao Xiong[2] , Mark R Segal[2*]

[1]Department of Bioengineering and Therapeutic Sciences,

[2]Department of Epidemiology and Biostatistics,

University of California, San Francisco, California, United States of America

[*]Corresponding author

Email addresses:

DC: daniel.capurso@ucsf.edu

HX: haoxiong@berkeley.edu

MRS: mark@biostat.ucsf.edu

Table S1. Number of sequence reads (or stable nucleosomes) for histone methylations and H2A.Z at each data pre-processing step.

In the columns labeled "Sequencing" through "Normalization", cells indicate the number of 25-base pair sequence reads [from Barski et al (2007)] that are retained at each data pre-processing step. In the final column, cells indicate the number of signal peaks that correspond to stable nucleosomes, identified using NPS.

**Histone Modification**	**Number of Sequence Reads**				**Number of Stable Nucleosomes**
	**Sequencing** *Barski et al, 2007*	**Alignment** *Barski et al, 2007*	**Deduplication** *Present study*	**Normalization** *Present study*	**Position-finding** *Present study*
H2A/H4R3me2s	25,128,493	7,357,597	7,140,521	4,330,278	1,854
H2A.Z	14,641,244	7,536,100	6,726,630	4,330,278	46,235
H2BK5me1	21,230,477	8,942,880	7,938,208	4,330,278	42,165
H3K4me1	37,461,698	11,322,526	9,921,429	4,330,278	74,544
H3K4me2	13,088,174	5,447,902	5,234,477	4,330,278	56,403
H3K4me3	39,872,596	16,845,478	13,344,169	4,330,278	64,453
H3K9me1	16,446,697	9,311,627	8,824,220	4,330,278	32,385
H3K9me2	19,712,420	9,782,127	9,411,727	4,330,278	282
H3K9me3	12,284,114	6,348,997	5,941,216	4,330,278	4,923
H3K27me1	20,481,466	10,047,279	9,705,780	4,330,278	2,132
H3K27me2	20,998,788	9,070,882	8,862,687	4,330,278	458
H3K27me3	28,475,252	8,970,141	8,632,665	4,330,278	689
H3K36me1	12,898,612	8,077,127	7,907,199	4,330,278	410
H3K36me3	30,015,905	13,572,575	12,362,519	4,330,278	14,495
H3K79me1	19,253,958	5,137,886	4,979,854	4,330,278	32,936
H3K79me2	4,341,935	4,712,875	4,448,350	4,330,278	84,571
H3K79me3	21,024,126	5,929,782	4,440,702	4,330,278	72,059
H3R2me1	16,465,425	9,560,224	9,195,984	4,330,278	602
H3R2me2a	14,743,869	6,521,560	5,953,869	4,330,278	571
H4K20me1	20,396,442	11,015,873	9,640,668	4,330,278	70,084
H4K20me3	18,380,292	5,720,089	4,330,278	4,330,278	17,962

Table S2. Number of sequence reads for histone acetylations at each data pre-processing step.

Cells indicate the number of 25-base pair sequence reads [from Wang et al (2008)] that are retained at each data pre-processing step.

**Histone Modification**	**Number of Sequence Reads**		
	**Sequencing** *Wang et al, 2008*	**Alignment** *Wang et al, 2008*	**Deduplication** *Present study*
H2AK5ac	9,260,603	3,442,542	3,400,544
H2AK9ac	4,228,439	2,070,246	1,882,070
H2BK5ac	7,635,650	3,330,268	3,066,338
H2BK12ac	9,438,261	3,615,226	3,515,350
H2BK20ac	7,868,594	4,083,727	3,929,081
H2BK120ac	7,693,057	3,444,551	3,280,474
H3K4ac	7,255,253	3,546,672	3,438,276
H3K9ac	9,357,424	3,950,661	3,726,987
H3K14ac	8,987,513	3,799,058	3,755,104
H3K18ac	9,227,062	4,249,604	4,046,345
H3K23ac	7,313,742	2,527,421	2,510,612
H3K27ac	8,529,409	3,433,165	3,198,818
H3K36ac	8,934,172	4,374,235	4,196,023
H4K5ac	8,829,494	4,118,574	4,020,280
H4K8ac	8,350,731	4,278,905	4,176,246
H4K12ac	6,641,441	3,677,187	3,602,609
H4K16ac	19,471,237	7,059,753	6,921,635
H4K91ac	5,087,302	3,191,156	3,016,564

Table S3. Percentage of sequence reads at each data pre-processing step that contain the motif "TCCATT".

Cells indicate the percentage of 25-base pair sequence reads that contain at least one occurrence of the motif "TCCATT". The column labeled "Position-finding" uses the 25-base pair sequence reads that contribute to the signal peaks of stable nucleosomes, identified using NPS.

**Histone Modification**	**Sequence reads (%) containing the motif "TCCATT"**				
	**Sequencing** *Barski et al, 2007*	**Alignment** *Barski et al, 2007*	**Deduplication** *Present study*	**Normalization** *Present study*	**Position-finding** *Present study*
H2A/H4R3me2s	3.70	2.96	2.52	2.51	56.79
H2A.Z	1.11	1.38	1.42	1.42	0.91
H3K9me3	4.41	2.48	1.95	1.95	9.24
H4K20me3	20.44	11.34	4.59	4.59	10.53
